# Esketamine alleviates hypoxia/reoxygenation injury of cardiomyocytes by regulating TRPV1 expression and inhibiting intracellular Ca^2+^ concentration

**DOI:** 10.1016/j.clinsp.2024.100363

**Published:** 2024-04-30

**Authors:** Ying Zhang, QuanMei Lu, HanChun Hu, ChunChen Yang, QiHong Zhao

**Affiliations:** a Department of Anesthesiology, The First Affiliated Hospital of Bengbu Medical College, Bengbu City, Anhui Province, China; b Department of Anesthesiology, The Second Affiliated Hospital of Bengbu Medical College, Bengbu City, Anhui Province, China

**Keywords:** Esketamine, TRPV1, Ca^2+^, Cardiomyocytes, Hypoxia/Reoxygenation Injury

## Abstract

**Objective:**

This study aimed to investigate the effect of Esketamine (ESK) on the Hypoxia/Reoxygenation (H/R) injury of cardiomyocytes by regulating TRPV1 and inhibiting the concentration of intracellular Ca^2+^.

**Methods:**

The H/R injury model of H9c2 cardiomyocytes was established after 4h hypoxia and 6h reoxygenation. H9c2 cells were treated with different concentrations of ESK or TRPV1 agonist capsaicin (10 μM) or TRPV1 inhibitor capsazepine (1 μM). Cell viability was detected by CCK-8 method, and apoptosis by flow cytometry. Intracellular Ca^2+^ concentration was evaluated by Fluo-4 AM. LDH, MDA, SOD, and GSH-Px were detected with corresponding commercial kits. TRPV1 and p-TRPV1 proteins were detected by Western blot.

**Results:**

After H/R, H9c2 cell viability decreased, apoptosis increased, intracellular Ca^2+^ concentration increased, LDH and MDA levels increased, SOD and GSH-Px levels decreased, and p-TRPV1 expression increased. ESK treatment rescued these changes induced by H/R. After up-regulating TRPV1, the protective effect of ESK on H/R injury of H9c2 cells was weakened, while down-regulating TRPV1 could further protect against H/R injury.

**Conclusion:**

ESK alleviates H/R injury of cardiomyocytes by regulating TRPV1 expression and inhibiting intracellular Ca^2+^ concentration.

## Highlights

• ESK treatment can increase H9c2 cell viability and reduce apoptosis and intracellular Ca^2+^ concentration.
• CAP treatment decreases H9c2 cell viability and increases apoptosis and intracellular Ca^2+^ concentration.
• TRPV1 upregulation decreases H9c2 cell viability and increases cell apoptosis and intracellular Ca^2+^ concentration.
• After overexpressing TRPV1, the protective effect of ESK on H/R injury of H9c2 cells is weakened.

## Introduction

The term Myocardial Ischemia-Reperfusion Injury (MIRI) refers to the fact that, following acute coronary artery occlusion, reperfusion can still cause a more serious injury than ischemia alone.^1^ Blood flow reperfusion may cause damage to non-involved myocardial cells as well as aggravate the damage to involved myocardial cells.^2^ Furthermore, some cytokines damage nonfocal organs with blood flow. Up to 50 % of myocardial injuries are caused by reperfusion, resulting in heart failure, arrhythmia, and even death.^3^

Transient Receptor Potential (TRP) is a widely distributed protein in the peripheral and central nervous system, which is divided into seven subfamilies: TRPA (Ankyrin), TRPC (Canonical), TRPM (Melastatin), TRPN (NOMPC-like) TRPML (Mucolipin), TRPP (Polycystin) and TRPV (Vanilloid).^4^ Transient Receptor Potential Vanilloid subfamily member-1 (TRPV1) is a cation channel belonging to the TRP family, which has dynamic selectivity for cations such as H^+^, Na^+^, Ca^2+^ and Mg^2+^.^5^^,^^6^ Intrathecal injection of the TRPV1 antagonist Capsazepine (CPZ) can inhibit TRPV1 activation and reduce the size of myocardial infarction.^7^ SUN et al.^8^ also believed that treatment of H9c2 cardiomyocytes with TRPV1 agonist CAP improves apoptosis, and treatment with CAP leads to an increase in intracellular CA^2+^ level, resulting in increased production of mitochondrial superoxide and depolarization of mitochondrial membrane potential. Esketamine (ESK), a right-handed version of ketamine, has been widely used in postoperative analgesia as a new analgesic.^9^, ^10^, ^11^, ^12^ ESK regulates autophagy and oxidative stress and has neuroprotective effects on traumatic brain injury.^13^ ESK can alleviate the activation of microglia in the ischemic cortex and reduce inflammatory cytokines. ^14^ In addition, ESK alleviates liver injury and oxidative stress by targeting the Nrf2/HO-1 pathway.^15^ However, what effect ESK has on MIRI remains unclear.

Therefore, the aim of this study was to investigate the effect of ESK on the Hypoxia/Reoxygenation (H/R) injury of cardiomyocytes by regulating TRPV1 and inhibiting the concentration of intracellular Ca^2+^.

## Materials and methods

### *Cell culture*

H9c2 is a rat embryonic ventricular myocardial cell line purchased from China Infrastructure of Cell Line Resource (Beijing, China). A humidified incubator containing 5 % CO_2_ at 37 °C was utilized to culture H9c2 cells in DMEM (Gibco).

### *H/R injury model*

The H9c2 cell injury model was induced by H/R. The cells were inoculated on 6-well plates and cultured using serum-free DMEM. The cells were then cultured in an incubator containing 1 % O_2_, 94 % N_2_, and 5 % NO for 4h, followed by an incubator containing 95 % air and 5 % NO for 6h. Normoxia was present in the control group.

### *Cell treatments*

H9c2 cells were treated with different concentrations of ESK (10 μg/mL, 20 μg/mL, 30 μg/mL) or the TRPV1 agonist CAP (10 μM) or the TRPV1 inhibitor CPZ (1 μM).

### *Cell transfection*

oe-NC, oe-TRPV1, si-NC, or si-TRPV1 were transfected into H9c2 cells using lipofectamine 3000.

### *CCK-8 assay*

H9c2 cells were inoculated in 96-well plates with 10 μL CCK8 reagent per well (Dojindo, Kumamoto, Japan). After 2h, microplate readers were employed to measure each well's absorbance (OD) at 450 nm in the dark. Blank wells contained only medium without cells, whereas control wells contained cells without treatment. Cell viability = (OD sample - OD blank)/(OD control - OD blank).

### *Flow cytometry*

H9c2 cells were analyzed for apoptosis using the Annexin V‐FITC/PI Apoptosis Detection Kit (Yeasen). Cells were washed twice with cold PBS, then suspended in 100 μL binding buffer with 5 μL Annexin V FITC and 10 μL PI, and allowed to incubate in the dark for 15 min. With a NovoCyte flow cytometer, cell suspensions were analyzed. Apoptotic cells ( %) = Annexin V-positive cells/total cells × 100 %.

### *Ca^2+^ measurements*

Using Fluo-4 AM staining, intracellular Ca^2+^ flux was measured. Cells were resuspended in Ca^2+^- and Mg^2+^-free PBS/glucose medium supplemented with 5 moL/L Fluo 4-AM and further detected in the dark at 37 °C for 30 min. In addition, cells were incubated for 30 more minutes to ensure that intracellular AM esters had completely de-esterified. With the aid of a flow cytometer, the fluorescence of Fluo-4 AM was measured.

### *LDH determination*

LDH in H9c2 cells was determined using the LDH detection kit (Jiancheng). At 450 nm, OD was measured in each well.

### *ELISA*

From H9c2 cells, supernatant samples were collected to measure SOD, MDA, and GSH-Px contents using MDA, SOD, and GSH-Px assay kits (#A001-3, A003-1, A005-1; Jiancheng).

### *RT-qPCR*

Cells were extracted with TRIzol one-step protocol (Invitrogen, USA) for total RNA content. By using UV detection and formaldehyde deformation electrophoresis, high-quality RNA was confirmed. qPCR was performed with qRT-PCR kit (Thermo Fisher). PCR primers (Table 1) were provided by Sangon (Shanghai, China). After the reaction, both amplification curves and dissolution curves were confirmed. TRPV1 gene expression was measured by the 2^−ΔΔCt^ method and normalized to GAPDH.

**Table 1:** RT-qPCR primers.

Genes	Primers (5’–3’)
TRPV1	Forward: GAAGCAGTTTGTCAATGCCAGCTA
	Reverse: AGGGTCACCAGCGTCATGTTC
GAPDH	Forward: GTCGGTGTGAACGGATTTG
	Reverse: TCCCATTCTCAGCCTTGAC

### *Western blot*

Assaying total protein from H9c2 cells using bicinchoninic acid assay (Thermo Fisher) measured the total protein concentration. Proteins were mixed with a sample buffer and boiled at 95 °C for 10 min. The protein (30 μg) was then separated by 10 % (w/v) electrophoresis and transferred to a PVDF membrane, followed by incubation with 5 % BSA for 1h and culture with primary antibodies p-TRPV1 (1:1000, AF8520, Affinity Biosciences) and GAPDH (1:1000, ab8245, Abcam) at 4 °C overnight. After TBST washing, the membrane was incubated with a secondary antibody for 1h, rinsed with TBST, and developed by enhanced chemiluminescence. Band signals were visualized by Bio-Rad Gel EZ imager (Bio-Rad, USA) and analyzed by Image J software to measure gray values.

### *Statistical analysis*

Analysis of the research results was conducted using SPSS 21.0 statistical software. Data were expressed as mean ± SD. The *t*-test was conducted to analyze the difference between two sets of data, and the one-way analysis of variance to analyze that between multiple sets. It was deemed statistically significant when *p* < 0.05 was defined. Three repetitions of all experiments were conducted.

## Results

### *ESK treatment can increase H9c2 cell viability and reduce apoptosis and intracellular Ca^2+^ concentration*

**Fig. 1:**
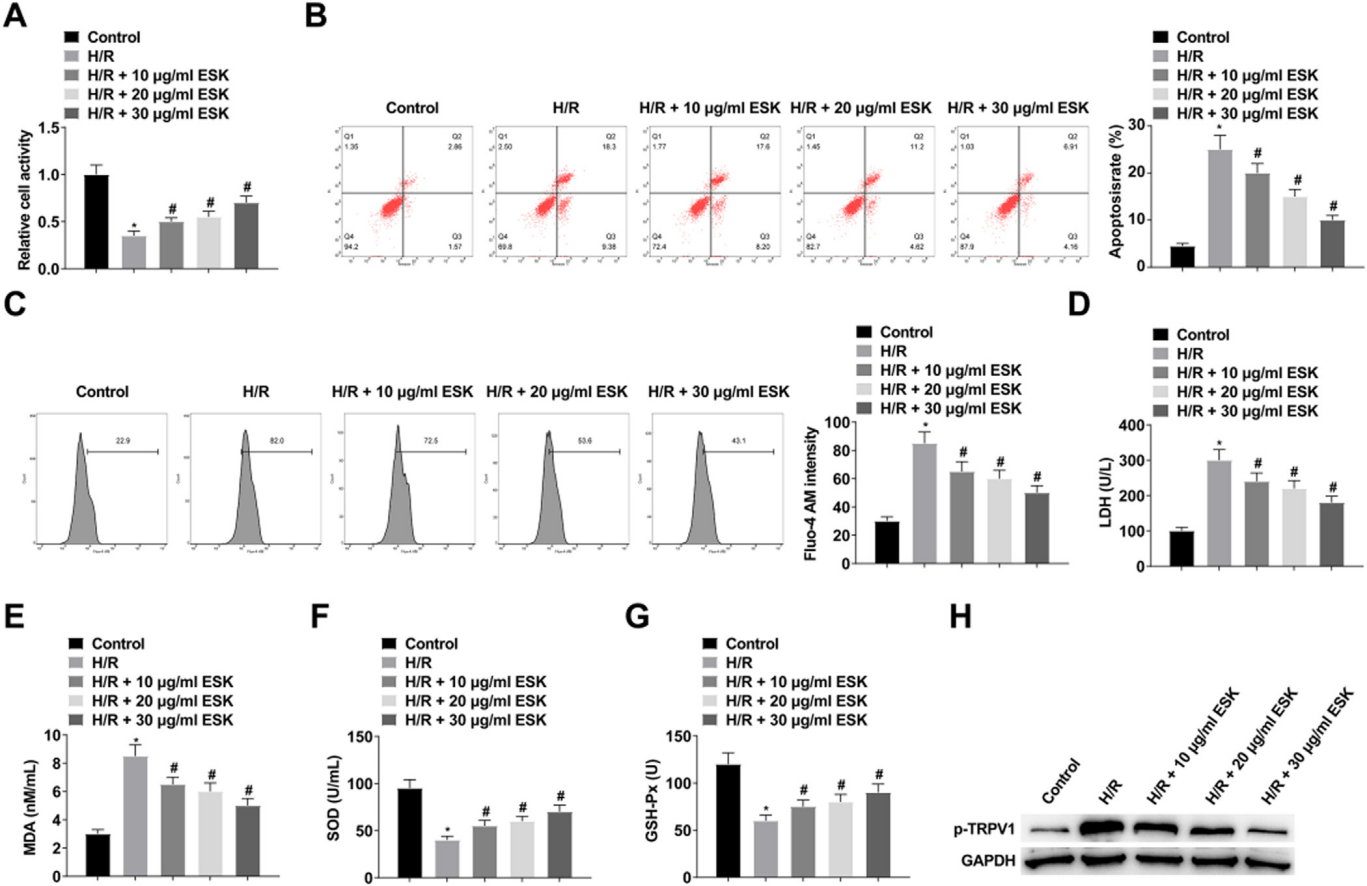
ESK treatment protects against H/R injury in H9c2 cells. (A) CCK-8 measured H9c2 cell viability; (B) Flow cytometry detected apoptosis; (C) Fluo-4 AM evaluated intracellular Ca^2+^ concentration; (D‒G) LDH, MDA, SOD and GSH-Px levels in H9c2 cells; (H) Western Blot measured p-TRPV1. Data are expressed as mean ± SD; (*) vs. Control group, *p <* 0.05; (#) vs. H/R group, *p <* 0.05.

The H/R injury model of H9c2 cardiomyocytes was established after 4 hours of hypoxia and 6 hours of reoxygenation. Cell viability was measured by the CCK-8 method, and the results showed that H/R decreased H9c2 cell viability (Fig. 1A). Apoptosis was detected by flow cytometry, and it was demonstrated that H/R promoted apoptosis (Fig. 1B). Intracellular Ca^2+^ concentration was assessed using Fluo-4 AM, which showed an increase in intracellular Ca^2+^ concentration after H/R (Fig. 1C). Corresponding commercial kits were used to detect LDH, MDA, SOD and GSH-Px levels. The results showed that H/R increased LDH and MDA levels, while decreased SOD and GSH-Px levels (Fig. 1D‒G). ESK treatment increased H9c2 cell viability and SOD and GSH-Px levels while reduced apoptosis, intracellular Ca^2+^ concentration, and LDH and MDA levels (Fig. 1A‒G), and 30 μg/mL ESK had the best protective effect on H9c2 cells. Therefore, 30 μg/mL ESK was selected for follow-up experiments. p-TRPV1 expression increased significantly after H/R, while ESK treatment inhibited p-TRPV1 expression (Fig. 1H).

### *CAP treatment decreases H9c2 cell viability and increases apoptosis and intracellular Ca^2+^ concentration*

**Fig. 2:**
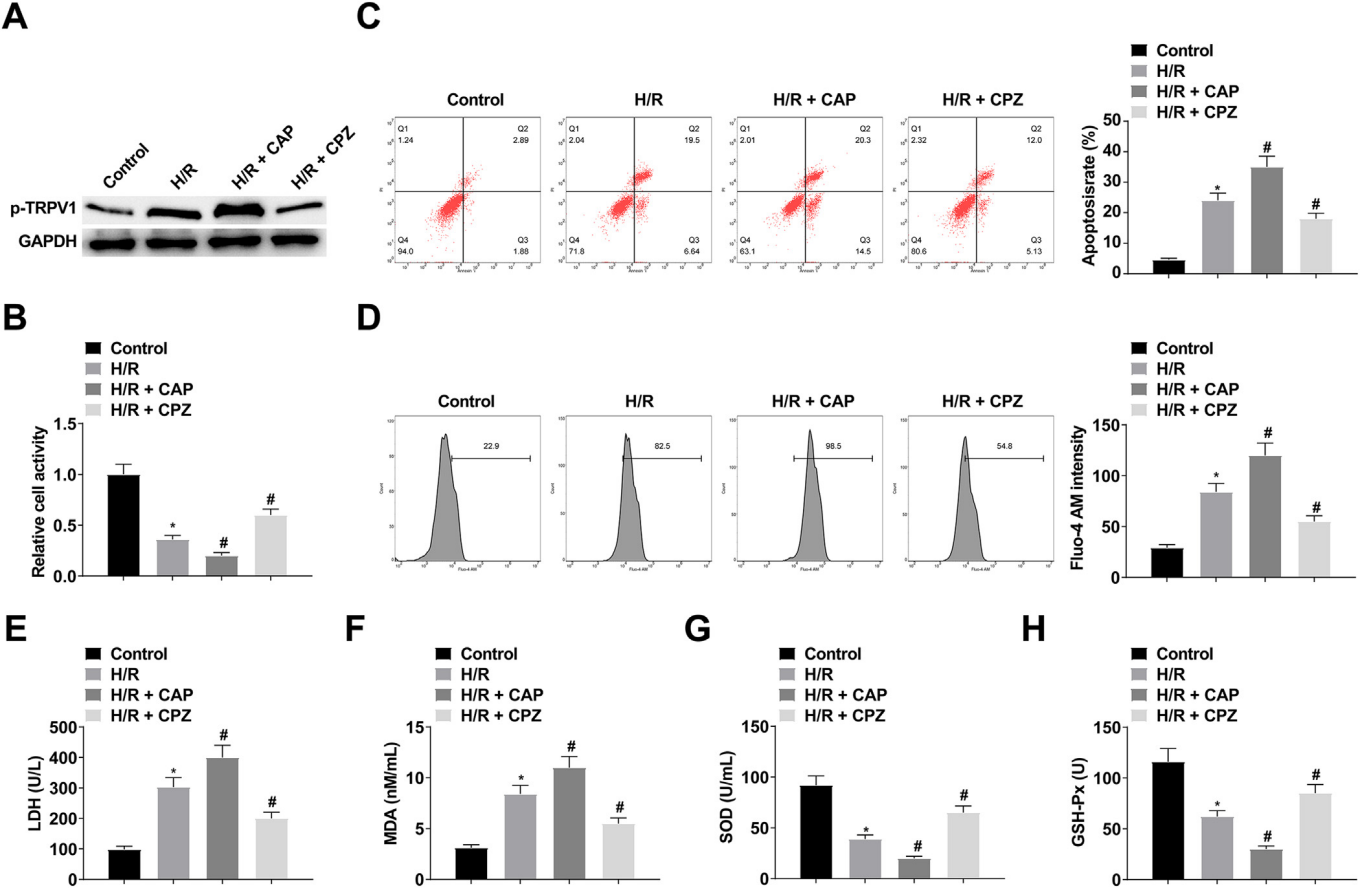
CAP treatment decreases H9c2 cell viability and enhances apoptosis and intracellular Ca^2+^ concentration. (A) Western Blot analysis of p-TRPV1 expression; (B) CCK-8 measured H9c2 cell viability; (C) Flow cytometry detected apoptosis; (D) Fluo-4 AM evaluated intracellular Ca^2+^ concentration; (E‒H) LDH, MDA, SOD and GSH-Px levels in H9c2 cells; Data are expressed as mean ± SD; (*) vs. Control group, *p <* 0.05; (#) vs. H/R group, *p <* 0.05.

To investigate whether TRPV1 is involved in the H/R process, H9c2 cells were treated with the TRPV1 agonist CAP (10 μM) or the TRPV1 inhibitor CPZ (1 μM). Western Blot results showed that CAP treatment could increase p-TRPV1 expression, while CPZ treatment could inhibit p-TRPV1 expression (Fig. 2A). CCK-8 results showed that H9c2 cell viability decreased after CAP treatment (Fig. 2B). Flow cytometry showed that CAP treatment promoted apoptosis (Fig. 2C). Intracellular Ca^2+^ concentration was assessed using Fluo-4 AM, which showed an increase in intracellular Ca^2+^ concentration after CAP treatment (Fig. 2D). CAP treatment significantly increased LDH and MDA, while decreased SOD and GSH-Px (Fig. 2E‒H).

### *TRPV1 upregulation decreases H9c2 cell viability and increases cell apoptosis and intracellular Ca^2+^ concentration*

**Fig. 3:**
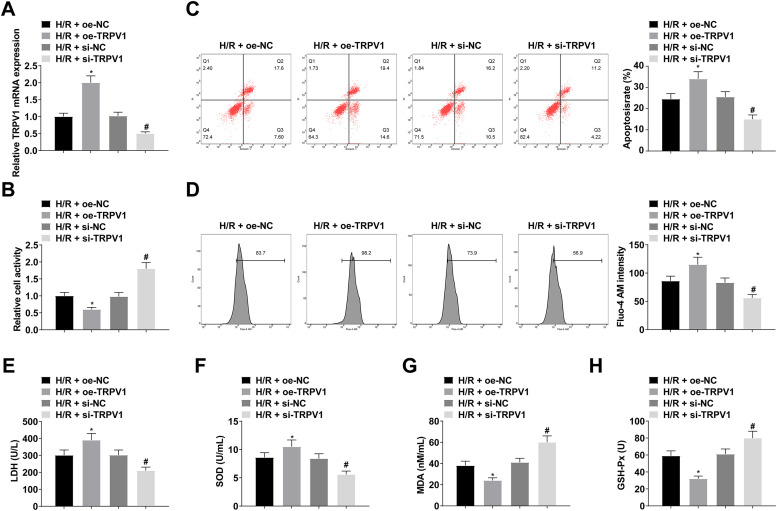
TRPV1 elevation decreases H9c2 cell viability and promotes apoptosis and intracellular Ca^2+^ concentration. (A) RT-qPCR and Western Blot verified transfection successfully; (B) CCK-8 measured H9c2 cell viability; (C) Flow cytometry detected apoptosis; (D) Fluo-4 AM evaluated intracellular Ca^2+^ concentration; (E‒H) LDH, MDA, SOD and GSH-Px levels in H9c2 cells; Data are expressed as mean ± SD; (*) vs. H/R + oe-NC group, *p <* 0.05; (#) vs. H/R + si-NC group, *p <* 0.05.

To further confirm TRPV1′s involvement in the H/R process, H9c2 cells were transfected with oe-NC, oe-TRPV1, si-NC, or si-TRPV1, and the transfection was verified by RT-qPCR (Fig. 3A). CCK-8 results suggested that H9c2 cell viability decreased after upregulating TRPV1 (Fig. 3B). Flow cytometry confirmed that upregulating TRPV1 promoted apoptosis (Fig. 3C). Intracellular Ca^2+^ concentration was enhanced after upregulating TRPV1 (Fig. 3D). Upregulating TRPV1 significantly increased LDH and MDA levels, while decreased SOD and GSH-Px levels (Fig. 3E‒H). However, the results after downregulating TRPV1 were all opposite (Fig. 3A‒H).

### *After overexpressing TRPV1, the protective effect of ESK on H/R injury of H9c2 cells is weakened*

**Fig. 4:**
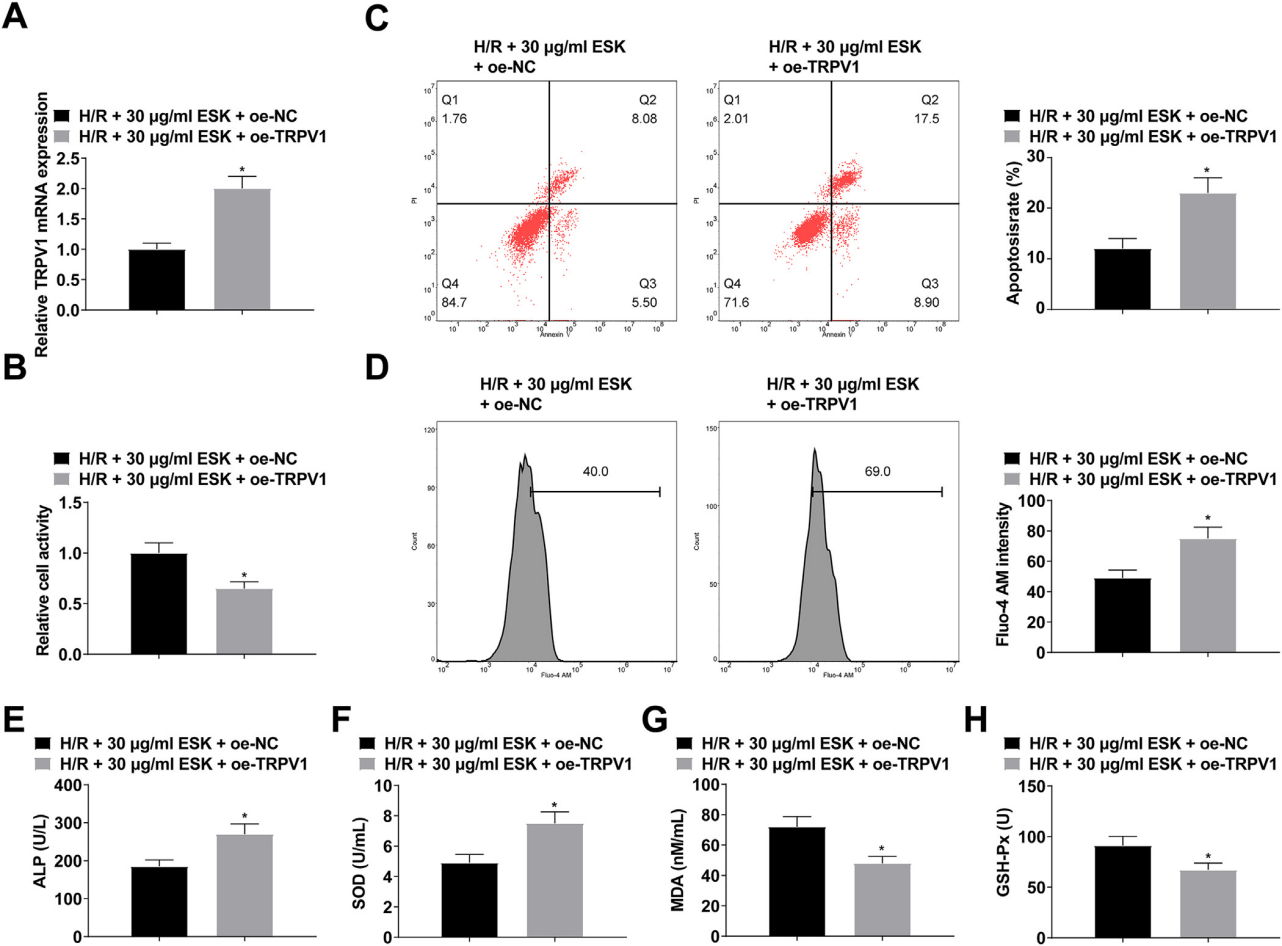
After up-regulation of TRPV1, the protective effect of ESK on H/R injury of H9c2 cells is weakened. (A) RT-qPCR verified transfection successfully; (B) CCK-8 measured H9c2 cell viability; (C) Flow cytometry detected apoptosis; (D) Fluo-4 AM evaluated intracellular Ca^2+^ concentration; (E‒H) LDH, MDA, SOD and GSH-Px levels in H9c2 cells; Data are expressed as mean ± SD; (*) vs. H/R + 30 μg/mL ESK + oe-NC group.

To verify the protective effect of ESK on H9c2 cells by regulating TRPV1 expression, H9c2 cells were treated with ESK and transfected with oe-TRPV1, which was successfully transfected by RT-qPCR (Fig. 4A). After overexpressingTRPV1, the promoting effect of ESK on H9c2 cell viability was weakened (Fig. 4B) as well as the suppressive effect on apoptosis (Fig. 4C). Also, the inhibition of ESK on intracellular Ca^2+^ concentration in H9c2 was reduced after upregulating TRPV1 (Fig. 4D). Further, elevating TRPV1 reduced the effects of ESK on LDH, MDA, SODm and GSH-Px levels in H9c2 cells (Fig. 4E‒H).

### *Suppressing TRPV1 can further increase H9c2 cell viability and reduce cell apoptosis and intracellular Ca^2+^ concentration*

**Fig. 5:**
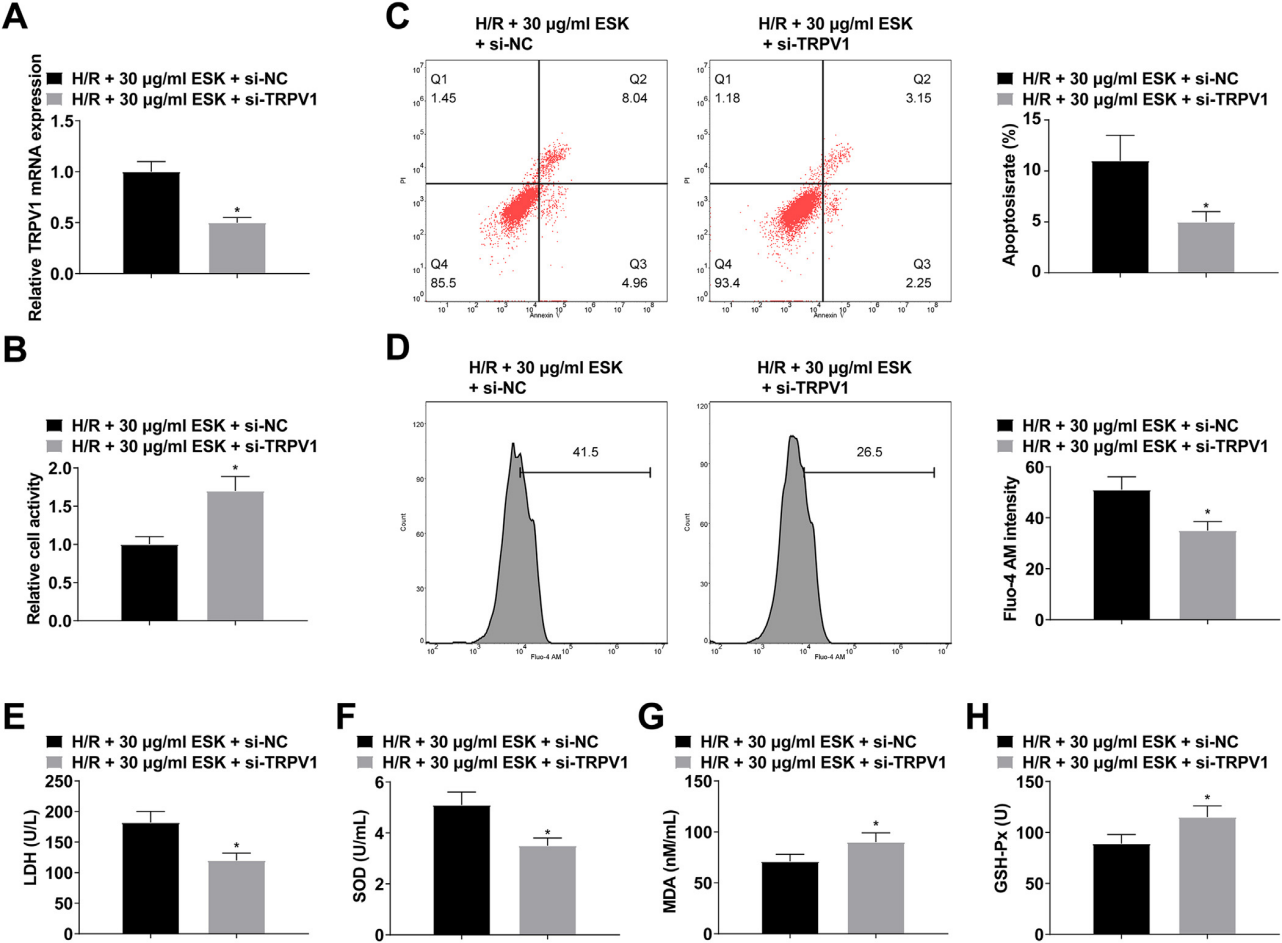
Suppressing TRPV1 can further protect against H/R injury. (A) RT-qPCR verified transfection successfully; (B) CCK-8 measured H9c2 cell viability; (C) Flow cytometry detected apoptosis; (D) Fluo-4 AM evaluated intracellular Ca^2+^ concentration; (E‒H) LDH, MDA, SOD and GSH-Px levels in H9c2 cells; Data are expressed as mean ± SD; (*) vs. H/R+30 μg/mL ESK + si-NC group.

H9c2 cells were treated with ESK and transfected with si-TRPV1, which was verified by RT-qPCR (Fig. 5A). Suppressing TRPV1 could further increase H9c2 cell viability and SOD and GSH-Px levels and reduce cell apoptosis and LDH and MDA levels (Fig. 5B‒H).

## Discussion

The main findings of this study are as follows: First, TRPV1 was activated in H/R injury of H9c2 cells; Secondly, CAP treatment activated the TRPV1 channel to promote H/R-induced apoptosis of H9c2 cells, while CPZ treatment had a myocardial protective effect, indicating that TRPV1 played a harmful role in H/R injury of H9c2 cells. Third, CAP treatment increased intracellular CA^2+^ levels, while CPZ treatment had the opposite effect, suggesting that CA^2+^ overload was involved in TRPV1-induced apoptosis. Fourth, upregulating TRPV1 decreased H9c2 cell viability and increased apoptosis and intracellular Ca^2+^ concentration, while downregulating TRPV1 had the opposite effect. Finally, ESK alleviated H/R injury by regulating TRPV1 expression and inhibiting intracellular Ca^2+^ concentration.

Cardiologists frequently encounter MIRI as a clinical pathological condition.^16^^,^^17^ In addition to acute myocardial infarction revascularization, it also occurs when blood flow is blocked, organ transplants are performed, and shock treatments during surgery.^18^^,^^19^ This condition is characterized by CA^2+^ overload, inflammation, oxidative stress, endoplasmic reticulum stress, mitochondrial dysfunction, and protease activation.^20^, ^21^, ^22^ As with other members of the TRP family, activation of TRPV1 can lead to a large influx of extracellular CA^2+^ ions.^23^, ^24^, ^25^, ^26^ Thus, induces apoptosis in a variety of intracellular events.^27^, ^28^, ^29^, ^30^ In this study, treatment with TRPV1 agonist CAP inhibited p-TRPV1 expression, and reduced H9c2 cell viability and SOD and GSH-Px levels while increased apoptosis, intracellular Ca^2+^ concentration, and LDH and MDA levels. The TRPV1 inhibitor CPZ had the opposite effect.

In H9c2 cells transfected with oe-TRPV1 or si-TRPV1, the authors further demonstrated the direct effects of TRPV1 on cardiomyocytes. In fact, upregulating TRPV1 decreased H9c2 cell viability and SOD and GSH-Px levels whereas increased apoptosis, intracellular Ca^2+^ concentration, and LDH and MDA levels. Downregulating TRPV1 had the opposite effect. These results confirm that TRPV1 plays a deleterious role in H/R-induced apoptosis.

Ketamine can bind to opioid receptors, NMDA receptors, cholinergic receptors, and monoaminergic receptors, inhibit local voltage-dependent ion channels, and have Ca^2+^ channel antagonism.^31^ In this study, ESK treatment can inhibit p-TRPV1 expression, increase H9c2 cell viability, reduce cell apoptosis, decrease intracellular Ca^2+^ concentration, suppress LDH and MDA levels, and activate SOD and GSH-Px activities. In addition, after upregulating TRPV1, the protective effect of ESK on H/R injury of H9c2 cells was weakened while downregulating TRPV1 could further protect against H/R injury.

Several limitations to the present study need to be mentioned. First, the downstream regulatory mechanisms of TRPV1 activation are unclear. Second, the apoptotic pathway has not been thoroughly studied, and in particular, the link between TRPV1 and caspase activation requires further investigation.

## Conclusion

ESK treatment can inhibit p-TRPV1 expression and enhance H9c2 cell viability and SOD and GSH-Px activities while suppressing apoptosis, intracellular Ca^2+^ concentration, and LDH and MDA levels. Elevating TRPV1 weakens the protective effect of ESK on H/R injury of H9c2 cells, while depleting TRPV1 could further protect against H/R injury. In conclusion, ESK alleviates H/R damage in cardiomyocytes by regulating TRPV1 expression and inhibiting intracellular Ca^2+^ concentration.

## Ethics statement

Not applicable.

## Consent to participate

Written informed consent was obtained from each subject.

## Consent for publishing

Written informed consent for publication was obtained from all participants.

## Availability of data and materials

The datasets used and/or analyzed during the present study are available from the corresponding author upon reasonable request.

## Authors’ contributions

Ying Zhang designed the research study. QuanMei Lu and HanChun Hu performed the research. ChunChen Yang and QiHong Zhao provided help and advice. Ying Zhang and Gao QiHong Zhao analyzed the data. Ying Zhang wrote the manuscript. QiHong Zhao reviewed and edited the manuscript. All authors contributed to editorial changes in the manuscript. All authors read and approved the final manuscript.

## Funding

Key Natural Science Project of Bengbu Medical College (2021byzd139).

## Declaration of competing interest

The authors declare no conflicts of interest.
